# First record of the ant subfamily Aenictinae (Hymenoptera, Formicidae) from Saudi Arabia, with the description of a new species

**DOI:** 10.3897/zookeys.228.3559

**Published:** 2012-10-11

**Authors:** Mostafa R. Sharaf, Abdulrahman S. Aldawood, Magdi S. El-Hawagry

**Affiliations:** 1 Plant Protection Department, College of Food and Agriculture Sciences, King Saud University, Riyadh 11451, P. O. Box 2460, Saudi Arabia; 2 Basic Sciences Department, Community College, Al-Baha University, Al-Baha, Saudi Arabia, P. O. Box 1598, Project: Survey and Classification of Agricultural and Medical Insects in Al-Baha Province

**Keywords:** *Aenictus*, taxonomy, Arabian Peninsula, Saudi Arabia, Al Sarawat Mountains, ants, Palaearctic region

## Abstract

The ant subfamily Aenictinae is recorded for the first time from the Kingdom of Saudi Arabia and for the second time from the Arabian Peninsula. A new species *Aenictus arabicus* **sp. n.**, is described from the worker caste. *Aenictus arabicus* belongs to the *Aenictus wroughtonii*-group and appears to be most closely related to *Aenictus rhodiensis* Menozzi, but can be easily distinguished from the latter by the following characters: overall smaller size; cephalic index (head width/head length) small; occipital corners in lateral view rounded; antennal scape when laid back surpassing approximately two-thirds of head length; funicular segments 2–8 each at least 2× as long as broad; subpetiolar process well developed; petiole and postpetiole distinctly imbricate; gaster and clypeus entirely yellow, teeth of mandibles reddish- brown. *Aenictus arabicus* was collected from leaf litter, next to a tree of *Psidium guajava* L. The new species also is similar to *Aenictus sagei* and *Aenictus wroughtonii*. Affinities and a key to related species of the species group are given.

## Introduction

The subfamily Aenictinae Emery, 1901, was elevated to the rank of subfamily by Bolton (1990), and includes a single genus, *Aenictus* Schuckard, 1840. The genus presently has 177 species and subspecific forms (Bolton 2012), distributed through the East Mediterranean, Afrotropical, Oriental, Indo-Australian, and Australian regions (Gotwald 1995, Brown 2000, Aktaç et al. 2004, and Jaitrong and Yamane 2012). Most of the species are tropical (Brown 2000), with terrestrial habitats, foraging in soil, leaf litter, most of the Southeast Asian species forage on the ground, and some on trees (e.g., Hirosawa et al. 2000) and hunting other ant species and termites (Gotwald 1995, Rosciszewski and Maschwitz 1994).

The subfamily Aenictinae is characterized by having:- a waist of two segments, with the spiracle of the postpetiole set behind the midlength of the tergite; all gastral spiracles circular; and the first gastral segment with a narrow, neck-like constriction behind the articulation with the postpetiole, 8-10 antennal segments, the frontal lobes reduced with the antennal sockets completely exposed, and the promesonotal suture absent (Bolton 1994). Species of *Aenictus* are generally small, monomorphic and yellow to dark brown. Members of the *Aenictus wroughtonii*-group share the following characters (Jaitrong et al. 2010): head narrow; posterior margin of head lacking collar; antennae long, 10-segmented; with long scape reaching or surpassing posterolateral corners of head; anterior clypeal margin bearing 5-10 denticles; mandibles subtriangular, with masticatory margin bearing 8-12 minute teeth in addition to a large apical tooth with a sharp apex; frontal carinae short; mesosoma narrow and elongate; legs thin and long; head entirely smooth and shiny; almost entire body clear yellow to yellowish brown.

Since Wilson’s (1964) revision, several authors have published taxonomic papers dealing with particular areas or species groups, e.g., Zhou (2001) (South China), Shattuck (2008) (Australia), Terayama (2009) (Taiwan), Zettel and Sorger (2010) (Borneo and the Philippines), Jaitrong and Yamane (2012) (Southeast Asia), and Bharti et al. (2012) (India). A catalogue of the 35 known Afrotropical members can be accessed on the website of Taylor (2012), with photographs also on Fisher’s Antweb.org (2012).

Ten species of *Aenictus* have been reported from the Palaearctic, nine of which are distributed in the Southwestern part of the region, Morocco in the west to Afghanistan in the east (Aktaç et al. 2004). Country records include *Aenictus rhodiensis* from Greece (Menozzi, 1936), Turkey (Aktaç et al. 2004), Iran (Radchenko and Alipanah 2004) and Israel (Kugler 1988); *Aenictus fuscovarius fuscovarius* Gerstäcker, *Aenictus fuscovarius sagittarius* Santschi and *Aenictus hamifer* Emery from Egypt (Sharaf 2006); and, an unidentified species from Yemen (Collingwood and van Harten 2001). The *Aenictus wroughtonii*-group was revised for the Oriental and Indo-Australian regions (Jaitrong et al. 2010) giving seven species. The new species, *Aenictus arabicus* belongs to this species group with resemblance to *Aenictus sagei* and *Aenictus wroughtonii* described by Forel from India.

Here the subfamily Aenictinae is recorded for the first time from Saudi Arabia and for the second time from the Arabian Peninsula. A new species, *Aenictus arabicus* sp. n., is described based on the worker caste. The queen and male are unknown. A key to the related species within the *Aenictus wroughtonii*-group is given.

## Materials and methods

The following abbreviations are used for particular morphological features and metrics:

TL Total length; the outstretched body length from the mandibular apex to the gastral apex.


HW Head width; the maximum width of the head in full-face view.


HL Head length; the maximum length of the head, excluding the mandibles.


CI Cephalic index (HW × 100/HL).


SL Scape length, excluding condylar bulb.


SI Scape index (SL × 100/HW).


ML Mesosoma length; the length of the mesosoma in lateral view, from the point at which the pronotum meets the cervical shield to the posterior base of the propodeal lobes or teeth.


PRW Pronotal width; the maximum pronotal width in dorsal view.


PL Petiole length; the maximum length of petiole measured in dorsal view, from the anterior margin to the posterior margin.


PW Petiole width; the maximum petiolar width measured in dorsal view.


PPL Postpetiole length; the maximum postpetiolar length measured in dorsal view.


PPW Postpetiole width; the maximum postpetiolar width measured in dorsal view.


All measurements are expressed in millimeters. Images were taken with a scanning electron microscope ((SEM) JSM-6380 LA).

### Depositories of type material

BMNH Natural History Museum, London, United Kingdom.


CASC California Academy of Science Collection, San Francisco, California, USA.


KSMA King Saud Museum of Arthropods, King Saud University, Riyadh, Kingdom of Saudi Arabia (Holotype depository).


MCZC Museum of Comparative Zoology, Harvard University, Cambridge, MA, USA.


MHNG Muséum ďHistoire Naturelle, Geneva, Switzerland.


NHMB Naturhistorisches Museum, Basel, Switzerland.


SEMC Division of Entomology (Snow Entomological Collections), University of Kansas Natural History Museum, Lawrence, Kansas, USA.


WMLC World Museum Liverpool, Liverpool, United Kingdom.


## Results

### Aenictus arabicus

Sharaf & Aldawood sp. n.

urn:lsid:zoobank.org:act:347C091D-1E98-4765-AEF5-10C4CACE8DDE

http://species-id.net/wiki/Aenictus_arabicus

Figs 1 –12

#### Holotype worker.

Saudi Arabia, Al Baha-Mukhwah Aqaba RD,19.IV.2012, 20.00000°N,41.43758°E, 1300 m, 19.IV.2012 (*M. R. Sharaf leg*.); deposited in the KSMA.

#### Paratype workers.

21 workers, same data as holotype; 1 deposited in **MHNG** (Dr Bernhard Merz); 1 deposited in **NHMB** (Mrs. Isabelle Zürcher-Pfander); 2 deposited in **CASC** (Dr Brian Fisher); 2 deposited in **MCZC** (Prof. E. O. Wilson); 2 deposited in **SEMC** (Prof. Michael S. Engel); 1 deposited in **WMLC** (Mr. Tony Hunter), 1 deposited in **BMNH** (Mr. Barry Bolton); the remaining specimens in **KSMA** (M. R. Sharaf).

#### Measurements.

**Holotype**: TL 3.0, HL 0.65, HW 0.52, SL 0.50, PRW 0.35, ML 0.95, PL 0.22, PW 0.15, PPL 0.17, PPW 0.15. Indices: SI 96, CI 80.

#### Paratypes.

TL 2.75-3.12, HL 0.60-0.72, HW 0.42-0.55, SL 0.40-0.52, PRW 0.20-0.35, ML 0.77-1.00, PL 0.22-0.27, PW 0.12-0.15, PPL 0.15-0.20, PPW 0.12-0.17. Indices: SI 77-104, CI 70-92. (n=11).

#### Description of worker.

Head entirely smooth and shining. In full-face view head distinctly longer than broad, with convex sides and nearly straight posterior margin; occipital corners in lateral view rounded; anterior clypeal margin with six small denticles; masticatory margin of mandibles armed with a large apical tooth followed by five smaller subequal teeth and a relatively larger basal tooth; when laid back, antennal scapes surpassing about two thirds of head length; all funicular segments at least twice as long as broad; terminal funicular segment about 2.5 × as long as the proceeding segment; mandibles dull with longitudinal striations; whole head dorsum and antennae with stiff scattered long hairs. Mesosoma in dorsal view broader anteriorly than posteriorly; promesonotum in profile distinctly convex, bearing many pairs of hairs; metanotal groove distinct; mesopleuron faintly but distinctly imbricate; propodeum bare or in some individuals with very sparse decumbent pubescence; propodeal dorsum long, about 4× as long as declivity; propodeum in profile slightly lower than promesonotum and almost flat dorsally; propodeal junction rounded. Petiole longer than broad in dorsal view with node clearly convex in lateral view; subpetiolar process triangular with convex ventral margin and blunt anteriorly. Postpetiole distinctly smaller than petiole, its node roundly convex, and its anteroventral edge sharp and bearing many hairs; both petiole and postpetiole distinctly imbricate and equipped dorsally with several pairs of backward directed long hairs. Gaster smooth and shining with abundant pairs of hairs. Color uniformly yellow.

**Figures 1–8.**
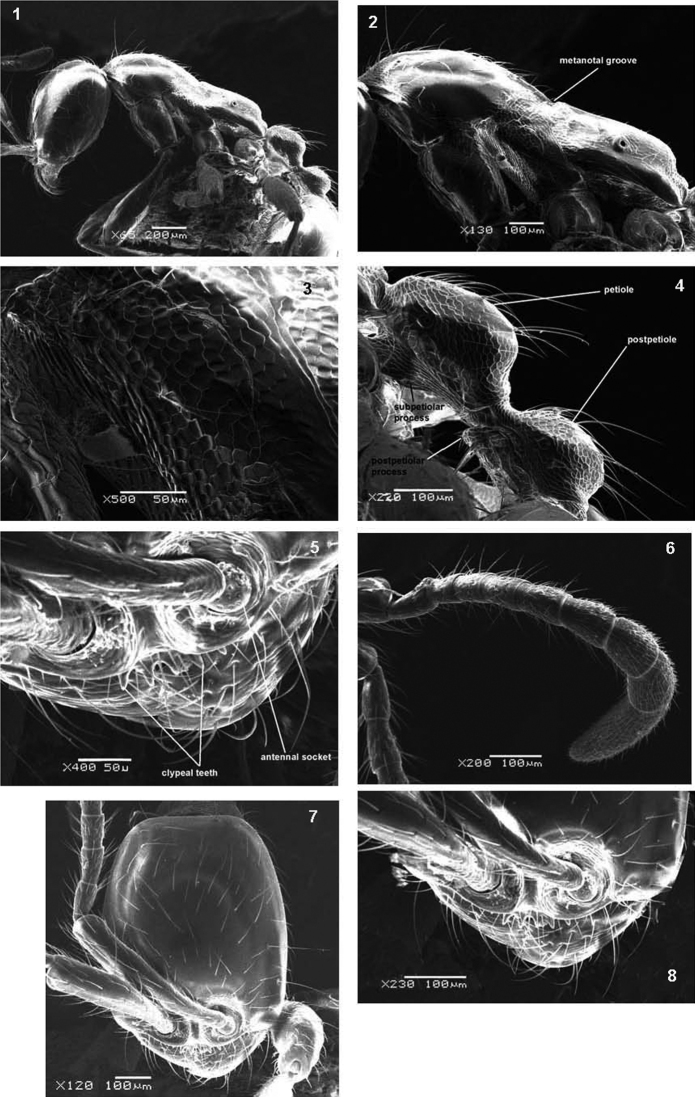
SEM of *Aenictus arabicus* sp. n. paratype **1** body in profile **2** mesosoma in profile **3** imbricate sculpture of mesopleuron **4** petiole and postpetiole in profile **5** antennal sockets and anterior clypeal margin **6** antenna **7** head in full-face view **8** anterior part of head.

**Figures 9–12.**
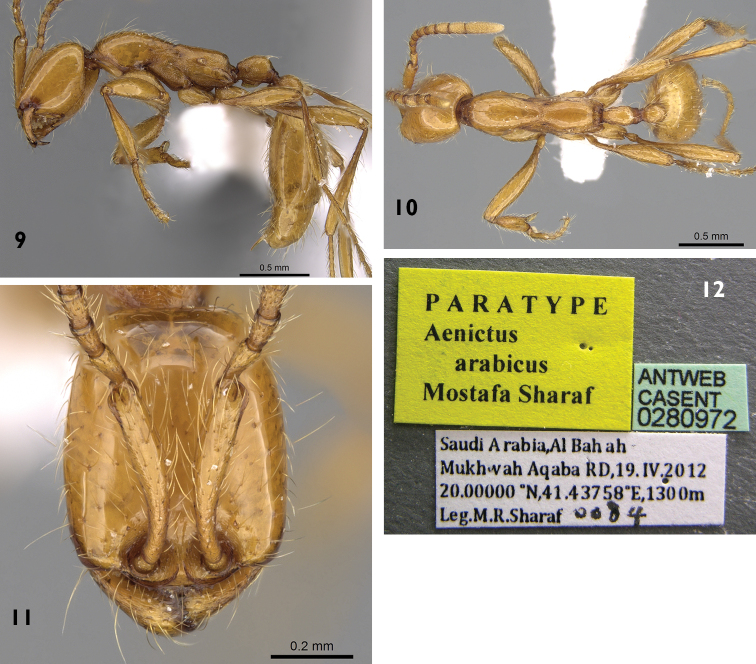
Automontage of *Aenictus arabicus* sp. n. paratype **9** body in profile **10** body in dorsal view **11** head in full-face view **12** label. ( CASENT0280972).

#### Etymology.

This species is named after the type locality.

## Discussion

**Affinities.** *Aenictus arabicus* is similar to *Aenictus rhodiensis* Menozzi, 1936 from Greece; and *Aenictus sagei* and *Aenictus wroughtonii* described by Forel from India. All the three species are members of the *Aenictus wroughtonii*-group as defined by Jaitrong et al. (2010) and sharing the following characters: head narrow, entirely smooth and shining; occipital margin lacking collar; antennae long, 10-segmented; anterior clypeal margin convex, rounded, with 5–10 denticles; mandibles subtriangular, with masticatory margin bearing 8–12 minute teeth in addition to a large apical tooth with a sharpe apex; mesosoma narrow and elongate; subpetiolar process weakly developed or almost absent; body clear yellow to yellowish brown.

Comparing *Aenictus arabicus* with *Aenictus rhodiensis*, both species have a similar general morphology, notably the shape of the mesosoma, petiole and postpetiole, a similar body pilosity; also both have a peculiar subpetiolar process which is somewhat wide and blunt anteriorly and the anterior clypeal margin is equipped with six small denticles. From the more accurate description in Aktaç et al.(2004), *Aenictus arabicus* can be separated readily from *Aenictus rhodiensis*. The former has a small relatively long, narrow head (HL 0.60-0.72, HW 0.42-0.55, CI 70-92) and long scapes, when laid back surpassing about two-thirds of the head length (SI 77-104) while the latter has a shorter head (HL 1.23, HW 1.02) and shorter scapes, which just surpass the midpoint of the head. *Aenictus arabicus* has a nearly straight posterior margin of the head whereas it is weakly concave in *Aenictus rhodiensis*. The funicular segments 2-8 are at least twice as long as broad in the former, while they are as long as broad in the latter. *Aenictus arabicus* has an entirely yellow clypeus and reddish-brown mandibular teeth while the sides of the clypeus and mandibular teeth are reddish brown in *Aenictus rhodiensis*. The gaster of *Aenictus arabicus* is entirely yellow, whereas in *Aenictus rhodiensis*, the middle of the third gastral tergite has two longitudinal brownish lines which diverge forward, sometimes reducing to small points. *Aenictus dlusskyi* Arnoldi, known only from the type series from Armenia, also resembles *Aenictus arabicus* but is of a similar size to *rhodiensis* (Aktaç et al. 2004).

Comparing *Aenictus arabicus* with the Asian species *Aenictus sagei* (CASENT0281958) and *Aenictus wroughtonii* (lectotype images are given in Jaitronget al.2010: 35), *Aenictus arabicus* has the anterior clypeal margin bearing six small denticles; *Aenictus sagei* has 9-10 denticles; whereas *Aenictus wroughtonii* has 8-10 denticles. In addition, *Aenictus arabicus* hasthe subpetiolar process well developed, triangular, with convex ventral margin and blunt anteriorly and body pilosity fewer and shorter; *Aenictus sagei* has a weakly developed subpetiolar process, with its ventral outline nearly straight; its anteroventral corners obtusely angulate and body pilosity distinctly long and abundant (length of the longest pronotal hair 0.20–0.25 mm, Jaitronget al.2010); whereas *Aenictus wroughtonii* has an undeveloped subpetiolar process, with its ventral outline feebly convex and without anterior angle and relatively sparse standing hairs which are shorter than in *Aenictus sagei*.

### Habitat and biology

**Figure 13.**
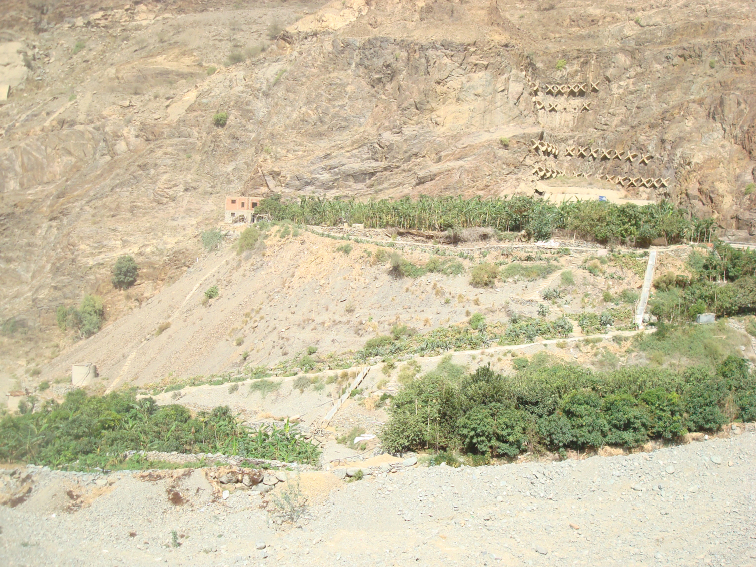
Type locality, Al Bahah, Mukhwah Aqaba RD. (photo M. R. Sharaf).

Al-Baha Province is divided by massive and steep rocky mountains into the lowland coastal plain to the west, known as “Tihama”, and the mountainous area ranging 1500 - 2450 m above sea level to the east, known as “Al-Sarat or Al-Sarah” which forms part of Al-Sarawat Mountains. The type locality (Fig. 13) is a small farm at the beginning of a narrow valley isolated between the mountains and the plain with a few native shrubs and trees at 1300 m. The farm is planted with *Annona squamosa* L. (Annonaceae), *Prunus persica* (L.), *Prunus Amigdalus* (Mill.) (Rosaceae), *Psidium guajava* L. (Family: Myrtaceae), *Zea mays* ssp. *mays* L. (Family: Poaceae), in addition to banana, and mango. The new species was found foraging on the ground under leaf litter and next to a tree of *Psidium guajava* L. The soil, at the time of collection was well saturated through irrigation and accumulation of organic matter.

The climate in Al-Baha Province is greatly influenced by its varying topography. It is generally moderate in summer and cold in winter with average temperatures ranging between 12–23 °C. In Tihama, the climate is hot in summer, warm in spring and mild in winter, with humidity ranging between 52%–67%, and a rainfall less than 100 mm annually. While in the mountainous area, Al-Sarah, The climate is greatly different from that in Tihama although they are separated by no more than 30 km. The weather is cooler in summer and winter due to its high altitude. Al-Sarah is exposed to the formation of clouds and fog, and this often happens in winter because of air masses coming from the Red Sea, accompanied by thunderstorms. In spring and summer, the climate is mild and pleasant. Also, rainfall is higher with falls in the range of 229–581 mm. The average rain falls throughout the whole province is 100–250 mm annually

Collingwood and van Harten (2001) recorded Aenictinae for the first time from the Arabian Peninsula based on an unidentified species, from workers collected in Yemen among leaf litter of banana plantations in Khamis Bani Sa’d and Lahj. The diagnostic characters given for this species indicate a similarity in general habitus to *Aenictus arabicus* but in two characters mentioned by them, a broadly emarginate head and unique crenulation of the anterior clypeal border, their species disagrees with the present new species. Future collecting in Yemen is needed to clarify the status of this taxon.

The presence of an *Aenictus* speciesin the Southwestern part of Saudi Arabia is not surprising as the area is regarded as being Afrotropical (Bodenheimer 1937; Nayman 1972; Sharaf et al. 2012; El-Hawagry et al. unpubl. data), and it is likely that more Afrotropical ants are awaiting discovery in the area.

Despite the Afrotropical nature of the type locality we found it important to give a key to the closely related species in the *Aenictus wroughtonii*-group.

### Key to species of the *Aenictus wroughtonii*-group related to *Aenictus arabicus* based on worker

**Table:** 

1	Subpetiolar process almost absent, anteroventrally not angulate (India)	*Aenictus wroughtonii*
–	Subpetiolar process present, its anteroventral corners angulate	2
2	Anterior clypeal margin bearing 9-10 denticles; subpetiolar process weakly developed (India)	*Aenictus sagei*
–	Anterior clypeal margin bearing six denticles; subpetiolar process well developed	3
3	Funicular segments 2–8 as long as broad; middle of third gastral tergite with two longitudinal brownish lines, sometimes reducing to small points; scapes when laid back just surpass the midpoint of head (Greece)	*Aenictus rhodiensis*
–	Funicular segments 2–8 at least twice as long as broad; gaster entirely yellow; scapes when laid back surpassing about two-thirds of head length (Saudi Arabia)	*Aenictus arabicus* sp. n.

## Supplementary Material

XML Treatment for Aenictus arabicus
